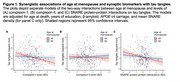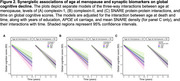# The interplay between synaptic integrity and age at menopause on Alzheimer's disease risk in women

**DOI:** 10.1002/alz70855_100373

**Published:** 2025-12-23

**Authors:** Madeline Wood Alexander, William G Honer, Rowan Saloner, Liisa AM Galea, David A. A. Bennett, Jennifer S Rabin, Kaitlin B Casaletto

**Affiliations:** ^1^ Sunnybrook Research Institute, Toronto, ON, Canada; ^2^ University of Toronto, Toronto, ON, Canada; ^3^ University of British Columbia, Vancouver, BC, Canada; ^4^ University of California San Francisco, San Francisco, CA, USA; ^5^ Centre for Addiction and Mental Health, Campbell Family Mental Health Research Institute, Toronto, ON, Canada; ^6^ Rush University, Chicago, IL, USA; ^7^ Division of Neurology, Department of Medicine, University of Toronto, Toronto, ON, Canada; ^8^ Hurvitz Brain Sciences Program, Sunnybrook Research Institute, Toronto, ON, Canada; ^9^ Memory and Aging Center, UCSF Weill Institute for Neurosciences, University of California, San Francisco, San Francisco, CA, USA

## Abstract

**Background:**

Early menopause increases Alzheimer's disease (AD) risk in women. Synaptic dysfunction incites and exacerbates AD progression. We investigated whether synergism between age at menopause and synaptic integrity influence AD neuropathology and cognitive trajectories in women.

**Method:**

We used autopsy and clinical data from the Rush Memory and Aging Project. Presynaptic proteins were measured across 6 cortical regions using immunoassays. We examined presynaptic terminal integrity via average levels of complexin‐I and complexin‐II, and presynaptic terminal functionality via average SNARE protein‐protein interactions. Age at spontaneous menopause was self‐reported. β‐amyloid and tau tangles were quantified with immunohistochemistry across 8 brain regions, and regional scores were averaged to produce summary measures. Cognition was assessed annually with neuropsychological tests, and standardized scores were averaged to compute a composite score of global cognition. Linear and mixed effects models tested interactive effects age at menopause and synaptic biomarkers on β‐amyloid, tau, and cognitive decline, adjusting for relevant covariates.

**Results:**

We included 268 women with spontaneous menopause (age at menopause=49.2±4.9; baseline age=83.7±5.9; age at death=90.9±5.9). For tau models, there were interactions of age at menopause with complexin‐I (β=0.070, *p* = .0002) and SNARE functionality (β=0.045, *p* = .02), but not complexin‐II (β=0.034, *p* = .13), such that earlier age at menopause exacerbated the associations of reduced synaptic integrity with elevated tau (Figure 1). There were no interactions of age at menopause with any synaptic biomarkers on β‐amyloid (*p*s>.15). For cognitive models, there were interactions between age at menopause and all three synaptic biomarkers (complexin‐I: β=‐0.008, *p* = .002; complexin‐II: β=‐0.006, *p* = .05; SNARE protein‐protein interactions: β=‐0.006, *p* = .01), such that women with earlier menopause exhibited stronger associations between reduced synaptic integrity and cognitive decline (Figure 2). Post hoc analyses suggested that the interactive associations of age at menopause and synaptic biomarkers on tau and cognitive decline were attenuated in women with a history of hormone therapy.

**Conclusion:**

In the setting of reduced synaptic integrity, women with earlier menopause show greater tau and steeper cognitive decline. These findings suggest that midlife endocrine processes or their sequelae modify the links between late‐life synaptic integrity and brain health outcomes. Interventions targeting both factors could promote resilience to AD in women.